# Ocrelizumab and ofatumumab comparison: an Italian real-world propensity score matched study

**DOI:** 10.1007/s00415-024-12360-x

**Published:** 2024-05-04

**Authors:** Aurora Zanghì, Giovanna Borriello, Simona Bonavita, Roberta Fantozzi, Elisabetta Signoriello, Stefania Barone, Gianmarco Abbadessa, Maria Cellerino, Vanessa Ziccone, Giuseppina Miele, Giacomo Lus, Paola Valentino, Sebastiano Bucello, Matilde Inglese, Diego Centonze, Carlo Avolio, Emanuele D’Amico

**Affiliations:** 1 Department of Medical and Surgical Sciences, University of Foggia, 71122 Foggia, Italy; 2 San Pietro-Fatebenefratelli Hospital, Rome, Italy; 3 Department of Advanced Medical and Surgical Sciences, University of Campania Luigi Vanvitelli, Naples, Italy; 4 IRCCS Neuromed, Pozzilli, Italy; 5 Second Division of Neurology, Department of Clinical and Experimental Medicine, University of Campania Luigi Vanvitelli, Naples, Italy; 6 Azienda Ospedaliera Universitaria “Mater Domini”, Catanzaro, Italy; 7 Department of Neuroscience, Rehabilitation, Ophthalmology, Genetics, and Mother-Child Health (DINOGMI), University of Genoa, Genoa, Italy; 8 Centro Sclerosi Multipla, UOSD Neurologia, ASP8 SR, P.O. Muscatello, Augusta, Italy; 9 Department of System Medicine, Tor Vergata University, 00133 Rome, Italy

**Keywords:** Ofatumumab, Ocrelizumab, Real-world comparison, NEDA3

## Abstract

**Background:**

The management of Multiple Sclerosis (MS) has undergone transformative evolution with the introduction of high-efficacy disease-modifying therapies (DMTs), specifically anti-CD20 monoclonal antibodies, such as ocrelizumab (OCR) and ofatumumab (OFA).

**Materials and methods:**

This is an independent retrospective cohort study in Relapsing MS (RMS) patients followed at eight Italian MS centers who initiated treatment with OCR or OFA in the participating centers and with at least 12 months on therapy. A generalized linear regression model inverse probability of treatment weight (IPTW) PS-adjusted was performed to evaluate the relationship between annualized relapse rate (ARR) and treatment groups. No evidence of disease activity-NEDA-3 at 12-month score was also collected. Safety profile of the investigated DMTs was recorded.

**Results:**

A total cohort of 396 RMS patients fulfilled the required criteria and were enrolled in the study. Out of them, 216 had a prescription of OCR and 180 of OFA. The mean follow-up was 13.2 ± 1.9 months. The estimated means for ARR did not show differences between the two groups, 0.059 for patients on OCR and 0.038 for patients on OFA (*p* = 0.185). The generalized regression model IPTW PS-adjusted did not reveal differences between patients on OCR and OFA (ExpB_OFA_ 0.974, 95%CI 934–1.015, *p* = 0.207). NEDA-3 at 12 months was experienced by 199(92.1%) patients on OCR and 170(94.4%) patients on OFA (*p* = 0.368). Generally, both therapies exhibit good tolerability.

**Conclusions:**

The treatment with OCR and OFA resulted in comparable control of disease activity with good safety profile. Our results need further validation in larger multicentre studies with long-term follow-up.

## Introduction

Multiple sclerosis (MS), a chronic and often debilitating neurological disease of the central nervous system (CNS), has been the subject of extensive research aimed at developing effective disease-modifying therapies (DMTs) [1].

The management of MS has undergone transformative evolution with the introduction of high-efficacy DMTs, specifically anti-CD20 monoclonal antibodies, such as ocrelizumab (OCR) and ofatumumab (OFA) [2–4].

OCR is a humanized anti-CD20 monoclonal antibody (from mouse) which binds to an overlapping epitope to that of rituximab and is used intravenously [5]. It was evaluated in two identically designed randomized clinical trials, OPERA I (NCT01247324) and OPERA II (NCT01412333), focusing on relapsing MS (RMS) [6, 7].

OFA is an anti-CD20, human monoclonal IgG1 antibody binding strongly to a distinct membrane epitope to rituximab and OCR [8]. OFA is the first type 1 immunoglobulin G1 kappa (IgG1κ) monoclonal antibody that is fully human, and it is administered subcutaneously. Its efficacy was assessed in two identically designed randomized controlled trials (RCTs), ASCLEPIOS I (NCT02792218) and ASCLEPIOS II (NCT02792231), involving patients with RMS [9].

Clinical trials have demonstrated the efficacy of both DMTs in reducing the annualized relapse rate (ARR) and disability progression, even when compared to established MS therapies, with generally good tolerability [6, 7, 9, 10].

However, available evidence is currently limited to a study simulating treatment comparisons of efficacy outcomes for OFA in ASCLEPIOS I/II versus OCR in OPERA I/II for the treatment of patients with RMS or to a network meta-analysis that assessed the efficacy of OFA against other drugs [11, 12]. While clinical trials investigated the safety and efficacy of these agents, there is a critical need to bridge the gap between controlled trial environments and the dynamic, multifaceted reality of real-world clinical practice. Efficacy and safety profiles are paramount considerations in selecting an appropriate MS treatment.

The primary objective of this study was to comparatively assess the efficacy of OCR and OFA therapies in a real-world cohort of patients with RMS. This evaluation used the ARR over the entire available follow-up period and the No Evidence of Disease Activity (NEDA-3) score at 12 months. As a secondary objective, the study examined the safety profile of the two DMTs and their impact on immuno-phenotype.

## Methods

### Setting and study design

This was an independent retrospective cohort study in RMS patients followed at eight Italian MS centers. Clinical and magnetic resonance imaging (MRI) data were collected prospectively by each MS center at routine clinic visits according to the national treatment guidelines [13]. Data were then collected retrospectively by chart review for this study. There was no interference with medical care received by the included patients.

### Study population

We included patients who (a) had a diagnosis of RMS according to 2017 McDonald criteria [14] and (b) initiated treatment with OCR or OFA between January 2022 and December 2022 in the participating centers and with at least 12 months on therapy.

We included patients with RMS naïve to any DMT or previously exposed to a moderate efficacy DMT (interferon beta products, glatiramer acetate, teriflunomide, and dimethyl fumarate) who switched to the index DMT for lack of efficacy.

Patients starting treatment with OCR or OFA were excluded if they were switching from high-efficacy DMTs (fingolimod, ozanimod, ponesimod, cladribine, natalizumab, ocrelizumab, alemtuzumab, rituximab, and cyclophosphamide).

We excluded data from patients who were lost to follow-up due to continuing treatment in another center.

During the considered time on therapy, all patients treated with OCR received infusions over a 6-month period.

Patients with progressive forms of MS receiving OCR were not included in the study as we considered comparison to OFA not to be relevant for this group.

**Fig. 1:**
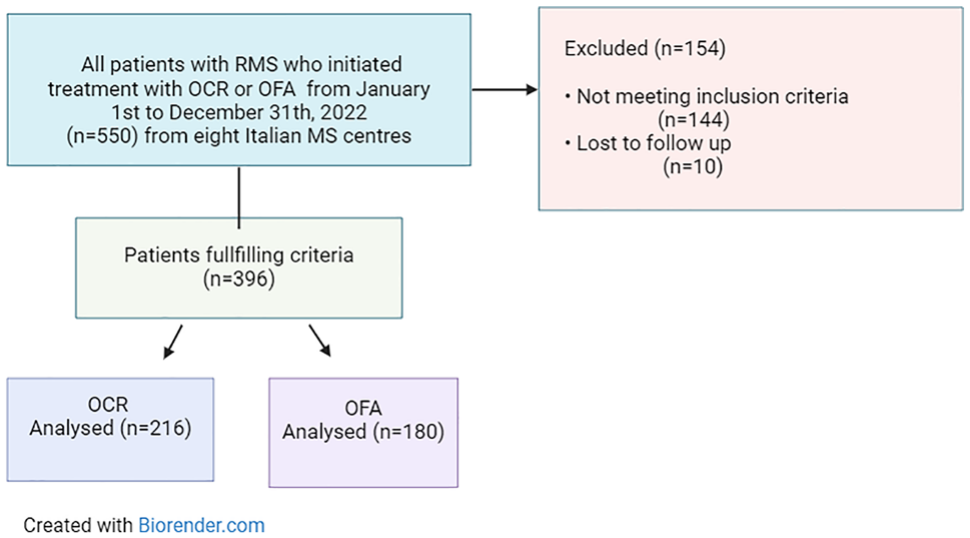
Flow chart of the study. OCR, ocrelizumab; OFA, ofatumumab

A flow chart of the study population is shown in Fig. 1.

### Procedures and covariate definition

Patients were treated in accordance with treatment procedures and guidelines approved by European and Italian Medicines Agencies.

In detail, OCR was administered at 600 mg/intravenous infusion, and the first 2 infusions—each of 300 mg—were given 2 weeks apart; subsequent 600 mg infusions were given every 6 months.

OFA was administered at an initial dosing of 20 mg by subcutaneous injection at weeks 0, 1, and 2, followed by subsequent dosing of 20 mg by subcutaneous injection once monthly starting at week 4.

Data were recorded retrospectively (including data until one year before index DMT) and prospectively (until the last available visit of follow-up) from the beginning of the investigated DMTs (the index date).

The data entry portal was iMed© software’s (iMed, Geneva, Switzerland). Data were extracted on November 30, 2023.

Disability was assessed by the Expanded Disability Status Scale (EDSS) by a neurostatus-certified MS specialist.

A cerebral MRI acquired within 12 months before the start of treatment was considered to assess disease activity in the year before the index date and the number of brain T2, and pre- and post-contrast T1 lesions was recorded. Follow-up MRIs to assess disease activity were acquired at 6 and 12 months after the start of the index DMTs.

Scans of all MS patients were performed in clinical routine, and all MRI were analyzed by a neuroradiologist with regard to new or enlarging lesions as well as to contrast-enhancing lesions. For each patient, the same scanner was used during follow-up, if possible, although this could not be achieved in all cases in clinical practice.

We classified the adverse events (AE) and severe adverse events (SAE) according to the European Medical Agency’s explanations. We regarded an AE as any medical occurrence in a subject who had been administered a pharmaceutical product, but lacking a necessary causal connection with the treatment [15].

SAE was defined as any AE that resulted in fatality, life-threatening inpatient hospitalization or extension of existing hospitalization, a persistent or significant incapacitation or substantial disruption of the ability to conduct normal life functions, a congenital anomaly/birth defect, or any occurrences that may necessitate medical or surgical intervention to prevent one of the outcomes listed in this definition.

Blood samples were collected in EDTA vials and processed within 2 h as a part of routine clinical practice. Flow cytometry acquisition was managed with the NAVIOS (Beckman Coulter) in each center. The Dura Clone IM B Cells kit was used for the evaluation of B cells.

We identified CD16 + CD56 + CD3- cells as Natural Killer cells. Among CD3 + CD45 + T cells, we identified the overall count of CD3 + CD45 + T cells and the subsets: CD3 + CD4 + (T helper) and CD3 + CD8 + (T cytotoxic). After gating on the CD19- positive cells, we considered CD19 + CD27 − IgD + (B-naïve) cells.

### Outcome measurements

The primary study outcome was the ARR on investigated drugs along with the available follow-up.

Secondarily, we calculated the proportion of patients with NEDA-3 at 12 months [defined as no relapses, no confirmed disability progression (CDP), and no active MRI lesions (both new or enlarged T2 lesions and contrast-enhancing lesions)]. Each of these subcomponents was also analyzed separately.

ARR was defined as total number of relapses divided by patient–months on therapy.

The relapse definition was standardized among Italian MS centers and was defined as the occurrence of new symptom(s) or the exacerbation of existing symptom(s) persisting for at least 24 h in the absence of concurrent illness or fever, occurring at least 30 days after a previous relapse.

CDP was defined as an increase in EDSS by ≥ 1.5 points for those with a baseline EDSS score of 0, by 1 or more points for a baseline score of ≤ 5.5, or by 0.5 points for a baseline score of > 5.5, which was sustained for 12 weeks or longer. EDSS recorded within 30 days after the onset of a relapse were excluded. MRI activity was considered new T1-gadolinium enhancing brain lesion and/or a new or newly enlarging T2 brain lesion.

Safety profile of the investigated DMTs was also investigated and reported.

Immunological subset was described before the beginning of the index DMT and after 6 months on therapy.

### Statistical analysis

Data are presented as proportion for categorical variables and mean (standard deviation) or median (interquartile range, IQR) for continuous variables.

The Kolmogorov test was used to verify data distribution. According to the latter, the parametric or nonparametric test was used.

In the first phase of the data analyses, a generalized linear mixed model with random intercepts was built using id center as the random effect [16]. Analysis of the covariance of the random intercept of ARR model did not reach significance (“ARR” model, Z-Wald 1.050, *p* = 0.294). The generalized linear model with fixed effects with the best statistical properties was then chosen according to the Akaike information criterion.

To consider the imbalance of the two groups, a propensity score (PS) was calculated as follows:

A logistic regression was performed to score all patients according to the treatment (OFA = 1 vs OCR = 0) used as independent variable and the following covariates at baseline: age, sex, disease duration, naïve/switch status, baseline EDSS, number of relapses in the year before index date, and MRI activity in the year before index date.

Inverse probability of treatment weight (IPTW) and the stabilized inverse probability of treatment weight (SIPTW) were also calculated. ORs and 95% CI were reported.

A generalized linear regression model IPTW PS-adjusted was performed to evaluate the relationship between ARR and treatment groups.

NEDA-3 score was compared using a contingency table.

Lymphocyte subsets were compared with ANOVA with Welch correction.

The statistical tests used are indicated in the figure or table legends. *p* values < 0.05 were considered significant.

Data were analyzed with IBM SPSS Statistics for Windows, Version 21.0 (IBM Corp. Released 2021, Armonk, NY, USA).

### Protocol approvals standard, registrations, and patient consents

The study protocol for the current analysis was also discussed and approved by the Scientific Committee, Comitato Etico Foggia (CE/14/2022). Each subject enrolled signed written informed consent to participate in the study. The current report does not contain any individual or identifying information.

### Data availability

Anonymized data will be shared by request from a qualified investigator for the sole purpose of replicating procedures and results presented in the report, provided that the data transfer is in agreement with EU legislation on the general data protection regulation.

## Results

From a total cohort of 550 patients, 396 fulfilled the required criteria and were enrolled in the study.

Out of them, 216 had a prescription of OCR and 180 of OFA (Fig. 1). Demographic and clinical characteristics of the whole cohort and groups are shown in Table 1**.**

**Table 1:** Baseline characteristics and cellular subsets of the enrolled cohort

Variables**	RMS (*n* = 396)	OCR (*n* = 216)	OFA (*n* = 180)	*p*-value*
Female n (%)	265 (66.9)	145 (67.1)	120 (66.7)	0.971
Age, year	37.9 ± 9.9	38.9 ± 10.3	37.1 ± 9.5	0.395
Smokers *n* (%)	138 (34.8)	76 (35.2)	62 (34.4)	0.819
Patient with Comorbidities n (%)	136 (34.3)	73 (33.8)	63 (35)	0.593
BMI (median, IQR)	23.8 (21.6–26.1)	24.4 (22.2–26.2)	23.6 (21.5–26.1)	0.745
Lag-time, months	15.9 ± 28.7	15.7 ± 28.1	15.9 ± 29.3	0.712
Disease duration, years	4.5 (1.5–13)	7 (2.2–15)	2 (0.5–10)	**0.004**
Patients naive to DMTs, *n* (%)	157 (39.6)	80 (37)	77(42.8)	0.210
Relapses in the year before index date	1.8 ± 0.7	1.8 ± 0.7	1.7 ± 0.6	0.359
Patients with MRI activity in the year before index date, *n* (%)	222 (56.1)	127 (58.8)	95 (52.8)	0.243
Baseline EDSS (median, IQR)	2.5 (2.0–4.0)	3.0 (2.0–4.5)	2.0 (1.5–3.0)	** < 0.001**

Lymphocytes, absolute counts (SD)^α^	(*n* = 186)	(*n* = 122)	(*n* = 74)	*p*-value^β^
Natural Killer CD16 + CD56 + CD3-cells	269.5 (170.7)	272.8 (196.1)	265.2 (130.2)	0.772
T CD3 + CD45 + cells	1410 (670)	1414.5 (723.2)	1404.5 (595.9)	0.926
T helper CD3 + CD4 + cells	848.6 (483.5)	861.5 (517.9)	829.5 (431.5)	0.709
T cytotoxic CD3 + CD8 + cells	464.7 (273.9)	456.1 (284.1)	474.2 (259.6)	0.660
B-naïve CD19 + CD27 − IgD + cells	222.3 (186.9)	217.9 (207.5)	265.2 (130.2)	0.679

BMI, Body Mass Index; EDSS, Expanded Disability Status Scale; MRI, magnetic resonance imaging; N. number; SD, standard deviation. *Via t test, Mann–Whitney U test or chi-squared test; **data are reported as the mean ± standard deviation unless otherwise specified

^α^ Normative values: CD3 + CD45+ T cells , 690–2540 cells/Ul, CD3 + CD4 + T helper, 410–1590 cells/Ul, CD3 + CD8 + T cytotoxic, 190–1140 cells/Ul; B CD19 + , 90–660 cells/Ul; CD16 + CD56 + CD3- Natural Killer, 90–590 cells/Ul.

^β^ via ANOVA with Welch correction. Statistically significant values are shown in bold

Patients on OCR had a higher EDSS value (median 3.0, IQR 2.0-4-5 vs 2.0, 1.5–3.0, *p* < 0.001) and longer disease duration in years than those on OFA (median 7, IQR 2.2–15 vs 2, 0.5–10, *p* < 0.001).

The mean follow-up was 13.2 ± 1.9 months. During the available follow-up, 18 patients relapsed: 13 (6%) on OCR and 6 (3.3%) on OFA (*p* = 0.029). All the relapses occurred during the first 12 months on therapy, and the mean time to first relapse was respectively 5.1 ± 3.2 and 4.6 ± 4.2 months (*p* = 0.799).

The estimated means for ARR did not show differences between the two groups, 0.059 for patients on OCR and 0.038 for patients on OFA (*p* = 0.185). The generalized regression model IPTW PS-adjusted did not reveal differences between patients on OCR and OFA (ExpB_OFA_ 0.974, 95% CI 0.934–1.015, *p* = 0.207).

MRI activity was observed in 4 (1.8%) patients on OCR and 5 (2.7%) patients on OFA (*p* = 0.451). All MRI activity was recorded during the first 12 months on therapy and time to MRI activity was respectively 6.5 ± 4.4 vs 7.2 ± 4.4 months (*p* = 0.077). Only one patient on OFA had a relapse and an associated MRI activity. No cases of CDP were reported in the two groups.

**Fig. 2:**
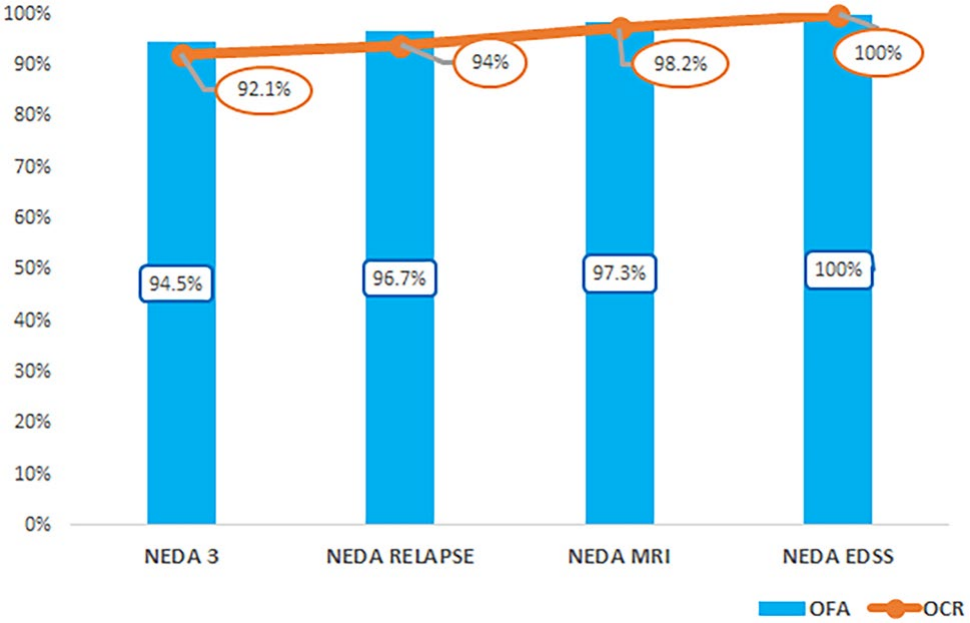
NEDA-3 at 12 months. NEDA-3, No Evidence of Disease Activity. OCR, ocrelizumab; OFA, ofatumumab

NEDA-3 at 12 months was experienced by 199 (92.1%) patients on OCR and 170 (94.5%) patients on OFA (*p* = 0.368). The rates for each NEDA-3 subcomponent are reported in Fig. 2.

### Safety profiles

During the observation period, the most reported AE in the OFA group was flu-like syndrome at the first administration (31, 17.2%) with early resolution (mean 6.5 ± 2.5 h). Upper respiratory tract infections were reported in 30 (13.8%) on OCR and 20 (11.1%) patients on OFA. The second most common AE in the OCR group was headache (14, 6.5%) (Table 2). No SAEs were reported in the investigated cohort.

**Table 2:** Adverse events in the enrolled cohort

Adverse events, n (%)	OCR (*n* = 216)	
	Upper respiratory tract infections	30 (13.9)
	Headache	20 (9.3)
	Urinary tract infections	9 (4.2)

Adverse events, n (%)	OFA (*n* = 180)	
	Flu like syndrome*	31 (17.2)
	Upper respiratory tract infections	20 (11.1)
	Urinary tract infection	6 (3.3)
	Urticaria*	4 (2.2)
	Headache	3 (1.7)

OCR, ocrelizumab; OFA, ofatumumab

^*^At the first administration

### Characterization of immunological subsets

Immunological subset characterization at time 0 and after 6 months on therapy was available in 186 patients (122 on OCR and 74 OFA).

According to a comparison between the two groups, patients on OFA had higher absolute counts of T CD3+CD45 +cells (1858.6 ± 893.7 vs 1068.5 ± 572.7, *p* = 0.037), and higher counts of T helper CD3+CD4 +  cells (1571.9 ± 2982 vs 710.4 ± 426.7, *p* = 0.012) and T cytoxic CD3+CD8 +  cells (684.3 ± 579.9 vs 398.1 ± 202.4, *p* < 0.001) after six months on therapy. Both groups maintained sustained B naïve cells suppression (0.8 ± 1.2 vs 2.5 ± 5.4, *p* = 0.091) and similar Natural Killer CD3-CD56 +  cells count (228.8 ± 95.8 vs 239.9 ± 134.1, *p* = 0.666).

## Discussion

In our real-world multicentre study, the treatment with OCR and OFA resulted in the control of disease activity assessed with ARR along the available follow-up and with 12-month NEDA-3.

OCR and OFA have been licensed as high-efficacy DMTs. However, no head-to-head studies have been provided, and to our knowledge, this is the first comparison in a real-world setting.

A recent network meta-analysis demonstrated that OFA was similar in efficacy to other highly efficacious monoclonal antibody therapies (i.e., alemtuzumab, natalizumab, and OCR) and ranked among the most efficacious DMTs in terms of reducing ARR in patients with RMS. The probability that OFA was the best treatment with respect to ARR was 28% [12].

Furthermore, an indirect treatment comparison was used to assess the comparative efficacy of OFA versus OCR while adjusting for differences in baseline characteristics between trials [11].

Here, ARR outcome after multivariate adjustment for baseline covariates, significantly favored OFA with a 40% reduction in relapse rates relative to OCR (RR: 0.60 [95% CI 0.43–0.84] [11]. Both prior to and following multivariate adjustment for baseline covariates, patients treated with OFA were significantly more likely to experience NEDA-3 between 48 and 96 weeks compared to those treated with OCR and the proportion of patients with Gd + T1 lesions was significantly reduced in patients treated with OFA compared to those receiving OCR [11].

However, results of an unanchored simulated treatment comparison are susceptible to residual confounding if patient characteristics are unbalanced across trials with respect to unmeasured prognostic factors and treatment effect modifiers. Undoubtedly, this recent analysis favoring OFA over OCR are suggestive of potential bias due in part to unmeasured characteristics, given the expectation that teriflunomide and IFN β-1a should have similar efficacy on the basis of a previous network meta-analysis.

Our data come from a real-world setting and have been corrected according to the PS methods, and, generally, both therapies exhibit good tolerability and comparable incidence of minor infections.

OFA administration was associated with a higher incidence of post-administration reactions, particularly tied to the initial dose, but the events subsided in the short-term period.

This was in accordance with data reported on registrative trials and with recently described real-world experiences discussed at ECTRIMS 2023 [17].

Moreover, data concerning the impact on T cell populations, despite inherent limitations stemming from a small sample size and restricted temporal observation, underscore the significance of TCD20 + cells [18–20]. Previous investigations have elucidated that TCD20 + lymphocytes, albeit constituting a modest proportion, undergo near-complete depletion with OCR likely attributable to its high-dose intravenous administration [19]. In contrast, a separate study on OFA revealed only a reduction without complete depletion of these lymphocytes [21, 22]. The data presented here, aligning partially with real-world observations previously published, suggest that the dosing regimen and administration method of OCR result in a more sustained depletion, while TCD20 + cells persist for a longer repopulation timeframe in patients undergoing OFA therapy.

These data require further confirmation for their interpretation. Certainly, CD8 lymphocytes have been implicated in the inflammatory process typical of MS, and the efficacy of OCR may lie in its high impact on TCD20 + lymphocytes, as supposed in previously published reports [23]. On the other hand, the increased CD4 lymphocytes in patients undergoing OFA therapy could be a consequence of the regulatory capabilities of these cells.

Indeed, it encourages further exploration into the long-term differential impact of the two treatments and whether this may affect therapeutic response.

Undoubtedly, the study exhibited several limitations. First, its observational nature inherently introduced numerous biases. While the PS serves as a valuable correction measure, it may not fully control for all potential confounders.

The analyses related to immuno-phenotype were not available for the entire patient cohort and were limited to the initial six months of follow-up. This is because the current protocol for OFA does not inherently include a scheduled analysis of immuno-phenotype, and often this analysis did not align temporally with the exact 12-month timeframe as in the case of OCR. Furthermore, despite using the same instrumentation across all centers, there exist unpredictable variabilities in the data.

Then, there was a limited follow-up period, so our results need further validation in larger multicentre studies with long-term follow-up.
